# Effect of Octenyl Succinic Anhydride (OSA) Modified Starches on the Rheological Properties of Dough and Characteristic of the Gluten-Free Bread

**DOI:** 10.3390/molecules26082197

**Published:** 2021-04-11

**Authors:** Jarosław Korus, Rafał Ziobro, Teresa Witczak, Kamila Kapusniak (Jochym), Lesław Juszczak

**Affiliations:** 1 Department of Carbohydrate Technology, Faculty of Food Technology, University of Agriculture in Krakow, 30-149 Kraków, Poland; rrkorus@cyf-kr.edu.pl (J.K.); rrziobro@cyf-kr.edu.pl (R.Z.); 2 Department of Engineering and Machinery for Food Industry, Faculty of Food Technology, University of Agriculture in Krakow, 30-149 Kraków, Poland; teresa.witczak@urk.edu.pl; 3 Department of Biochemistry, Biotechnology and Ecotoxicology, Faculty of Science and Technology, Jan Dlugosz University in Czestochowa, 42-200 Częstochowa, Poland; k.kapusniak@ujd.edu.pl; 4 Department of Food Analysis and Evaluation of Food Quality, Faculty of Food Technology, University of Agriculture in Krakow, 30-149 Kraków, Poland

**Keywords:** OSA starch, gluten-free bread, rheology, texture, staling

## Abstract

The study focused on the influence of starch modified by octenyl succinic anhydride (OSA) on the rheological and thermal properties of gluten-free dough containing corn and potato starch with the addition of pectin and guar gum as structure-forming substances. The starch blend used in the original dough recipe was partially (5% to 15%) replaced with OSA starch. The rheological properties of dough samples were determined, and the properties of the resulting bread were analyzed. It was found that the dough samples behaved as weak gels, and the values of storage and loss moduli (G′ and G″, respectively) significantly depended on angular frequency. Various shares of OSA starch in recipes modified dough in different ways, causing changes in its rheological characteristics. The introduction of OSA starch preparations resulted in changes in the bread volume and physical characteristics of the crumb. All the applied preparations caused an increase in bread porosity and the number of pores larger than 5 mm, and there was a parallel decrease in pore density. The presence of OSA starch preparations modified bread texture depending on the amount and type of the applied preparation. The introduction of OSA starches in gluten-free bread formulation caused a significant drop in the enthalpy of retrograded amylopectin decomposition, indicating a beneficial influence of such type of additive on staling retardation in gluten-free bread.

## 1. Introduction

In the absence of wheat-related proteins in gluten-free products, starch becomes their principal structure forming component, which has a strong influence on their appearance and texture, and thus consumer acceptance [1,2]. Corn, rice, and potato starches are most commonly used in such products, as they originate from gluten-free raw materials [3,4,5]. The properties of products in which starch is responsible for structure formation largely depend on its origin. Both botanical source and applied modifications significantly influence the microstructure and rheological properties of dough, water retention, and the final quality of starch-based foods [4,6,7]. Even starches derived from different varieties of the same plant species may have different physicochemical and functional properties [8].

Gluten-free products are often based on native starch, which is regarded as a food component, but they could also contain chemically modified starches with a status of approved additives. Their presence influences the water absorption and rheological properties of the dough as well as the texture and staling of bread crumb [9]. Chemically modified starches stabilize the crumb structure, diminish the retrogradation tendency, and thus delay bread staling. On the other hand, the excessive addition of some types of starch (e.g., cross-linked) could result in an unacceptable increase of crumb hardness [10].

Among possible additives applicable in gluten-free bakery products, esters of starch with octenyl succinic acid (OSA) could be especially interesting [11]. This type of modified starch is obtained in a reaction with octenyl succinic anhydride, and the esters are hydrocolloids with amphiphilic properties. New hydrophobic groups introduced during esterification, together with structural changes in starch granules lead to a decrease in pasting temperature and enthalpy, better swelling, lower digestibility, and increased viscosity and clarity of the gels. OSA starches find a broad range of applications in food industry, mainly as emulsifiers, encapsulating agents, and fat replacers [12,13].

The chemical modifications of starch can be accompanied by the action of various physical treatments. The most important is the pregelatinization combined with drying using roller or spray dryers. The pregelatinization of natural starch or its chemically modified derivatives, e.g., OSA starches, improves water binding. Starch derivatives obtained by this method swell easily in cold water. This may have a positive effect on the structure and properties of the dough and the retention of fermentation gases [14]. Another modification of OSA starches may be their partial enzymatic hydrolysis. The preparations obtained in this way contain polymers of reduced molecular weight. Thus, they dissolve better in water and stabilize the emulsion systems.

OSA starches are used in baking mostly as ingredients of traditional, wheat-based products. Dapčević-Hadnađev et al. [14] found that the effects of their incorporation in dough recipes depend on the physical form of their preparations. The use of intact granules of esterified starch causes an increase of water absorption and resistibility to deformation. At the same time, hydrolyzed OSA starch decreases water binding and enhances dough susceptibility. According to the authors, the presence of OSA starch in bread formulation positively impacts its characteristics. Dapčević-Hadnađev et al. [15] observed that the introduction of OSA starch may result in a decreased stability of wheat dough, but it improves bread volume and texture. Pojić et al. [16] observed that OSA starch absorbs more water and, in this way, it increases dough yield. Akram et al. [17] stated that OSA starch causes an increase in the viscosity of wheat flour dispersion during pasting. Pregelatinized OSA starch was also applied to improve the quality of barley bread [18]. It was found that such addition significantly influences the bread volume and crumb characteristics. OSA starch was also applied as an emulsifier in gluten-free bread based on rice and hemp flour [11]. It was noticed that the presence of OSA starch results in an increase of volume and improvement of texture properties. OSA starch preparations were also used as fat replacers in bakery products [19].

While there are a lot of data concerning the use of OSA starches to improve the quality of wheat-based bakery products, less information could be found about the use of these starches in the preparation of gluten-free bakery products, especially those based on native starch. Therefore, the aim of this study was to evaluate the effect of different OSA starch preparations: waxy corn starch sodium octenyl succinate, pregelatinized waxy corn starch sodium octenyl succinate, and starch hydrolyzed and spray-dried waxy corn starch sodium octenyl succinate, on the structure and aging of gluten-free bread. The influence of partial replacement (5% to 15%) of the basic raw material, i.e., corn and potato starch mixture, by OSA starch preparations on the rheological properties of the dough, selected quality parameters of the obtained bread, and its staling rate was evaluated.

## 2. Results and Discussion

### 2.1. Rheological Properties of the Dough

**Figure 1:**
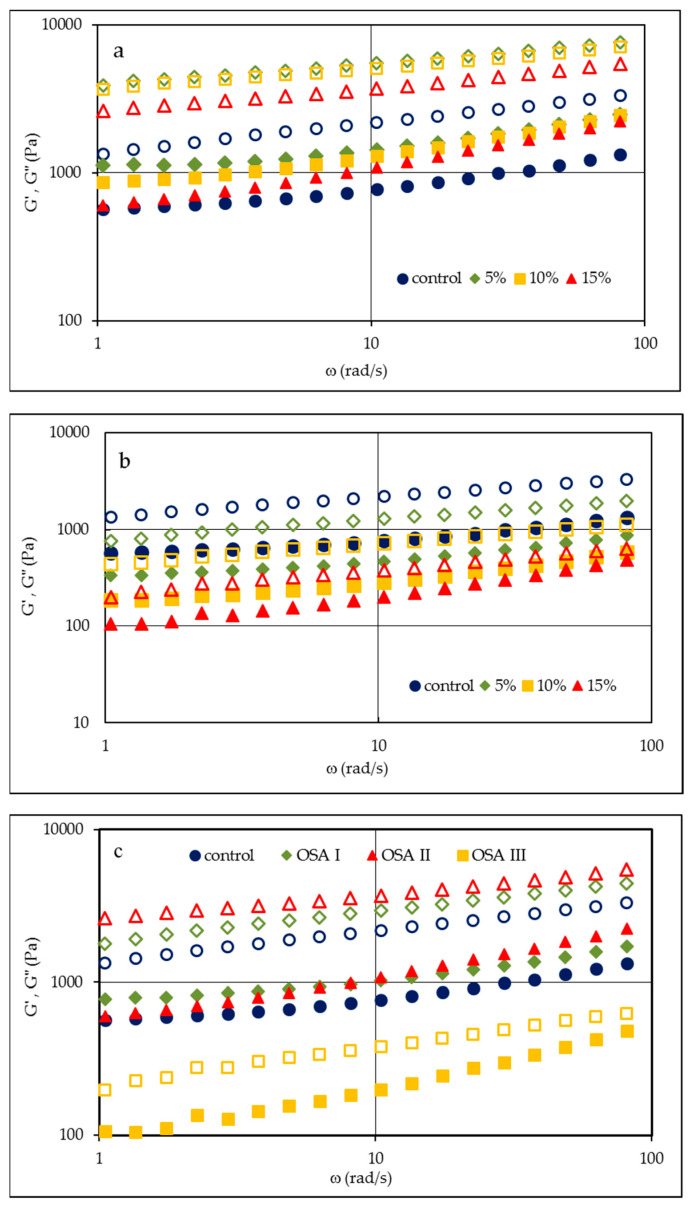
Mechanical spectra of control dough and samples of dough with octenyl succinic anhydride (OSA) starch II (**a**) and III (**b**) with different substitution levels of base starches and respective plots of all OSA starches with the highest substitution level (15%) of base starches (**c**); G′-filled symbols, G″-empty symbols. OSA I starch—waxy corn starch sodium octenyl succinate; OSA II starch—pregelatinized waxy corn starch sodium octenyl succinate; OSA III starch—hydrolyzed and spray-dried waxy corn starch sodium octenyl succinate.

The structure of the dough based solely on swollen starch granules and non-starch hydrocolloids is much weaker than when gluten-containing flours are used; its consistency is more viscous and less elastic [5]. Therefore, new formulations and technological processes have been developed that could improve the structure, rheological properties, and gas-retaining properties of gluten-free dough, which could result in a well aerated, porous structure of bread crumb. The modifications of bread recipe, addition or replacement of some dough components, are usually connected with changes in the rheological characteristics of the dough, which could be monitored by viscoelastic properties. Gluten-free dough under small deformations behaves as a weak gel [20,21]. Basic methods that could be applied to describe its properties are sweep frequency and creep and recovery tests. Figure 1a,b demonstrate the changes in storage (G′) and loss (G″) moduli, reflecting the share of elastic and viscous characteristics, in relation to angular frequency for the control and gluten-free doughs in which 5, 10, or 15% of the starch mix was replaced with OSA starches II and III, respectively. Figure 1c represents a similar dependence for control and all samples in which the 15% replacement level was applied. In all the cases, the values of the storage modulus were higher than the loss modulus G′ > G″, which demonstrates the prevalence of elastic properties over viscous ones and confirms earlier results about the viscoelastic character of starch-based gluten-free dough [21,22].

The obtained mechanical spectra show the varying influence of OSA starches on the viscoelastic properties of the dough. An addition of OSA starch I, which was unmodified after esterification, caused an increase in the values of G′ and G″: the larger they were, the higher was the concentration of this starch in the dough (curves not shown). On the other hand, the presence of OSA starch II, which was pregelatinized, affected the values of G′ and G″, and their highest level could be observed for the lowest 5% share of OSA starch. A further increase in OSA starch concentration caused a decrease in moduli; however, even at the highest level of replacement, their values were still higher than those registered for the control sample. The use of OSA starch III, which was hydrolyzed and spray-dried, significantly reduced the values of both moduli: the larger they were, the greater was the share of its preparation in the bread recipe, which may be related to the lower swelling capacity of this preparation. At the identical 15% replacement level (Figure 1c), a significant increase of moduli with angular frequency could be seen in the case of OSA II starch, followed by OSA I starch. The presence of OSA III starch significantly reduced the values of moduli, indicating a weakening of dough structure and a drop in both viscous and elastic properties. A significant increase could be seen in the values of the G′ and G″ moduli in the case of the dough with OSA II preparation, i.e., the pregelatinized OSA starch. It resulted from its high swelling capacity, which strengthens the structure of the dough. An increase in the elasticity and viscosity of the dough, but to a much lesser extent, was also observed for the sample with OSA I starch. This sample showed a lower swelling capacity. On the other hand, the introduction of hydrolyzed and spray-dried OSA starch (OSA III) into the recipe significantly weakened the structure of the dough. This may be due to the reduction of the molecular weight of starch polymers due to hydrolysis and thus to a lower structure-forming capacity.

The parameters of the power models describing the resulting experimental curves are summarized in Table 1. A two-factor variance analysis showed that both the type of OSA starch preparation used and the level of its contribution to the formulation significantly (*p* < 0.05) affect the values of these parameters. The values of the K′ parameter indicating the initial storage modulus G′ increased with the rising share of OSA I starch, although its lowest 5% level had no significant impact here. In the case of OSA II starch, the K′ values were the highest, although they decreased as the starch share in the recipe increased. In turn, in the presence of OSA III starch, the K′ values were reduced, and their drop was the greater the higher was its concentration. The values of parameter n′ indicating the relationship of G′ modulus to angular speed did not change significantly as a result of the addition of OSA I and II starch, with the exception of the highest 15% replacement level. On the other hand, when OSA II starch was introduced into the recipe, the values of n′ decreased significantly, indicating an increase in the dependence of G′ on frequency.

**Figure 2:**
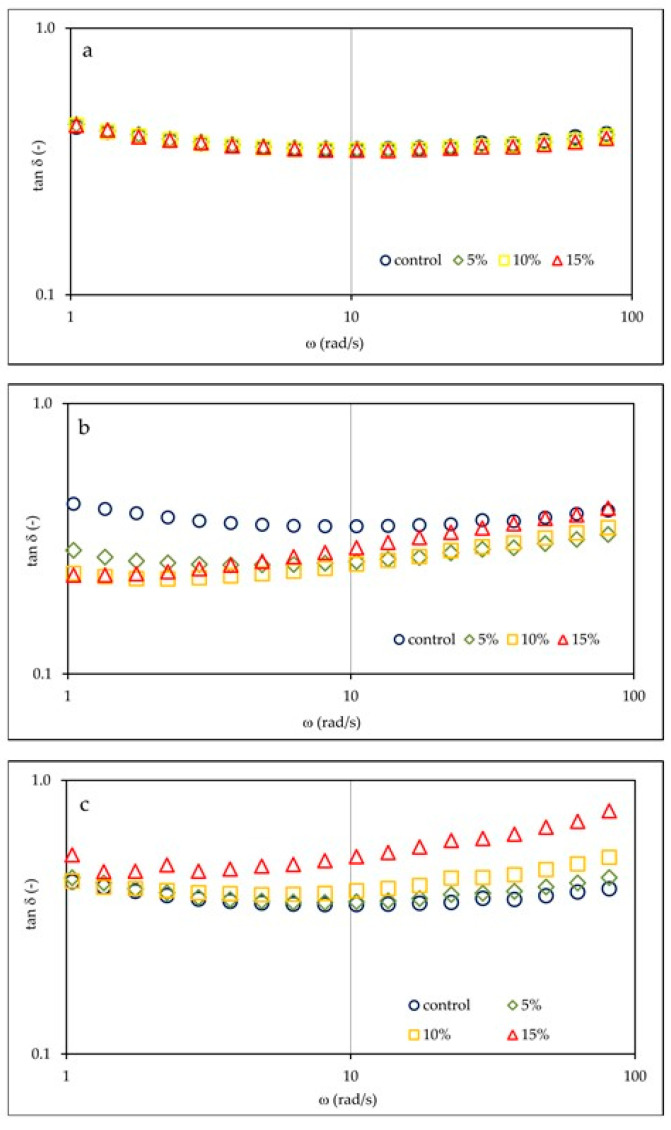
Shift angle tangent (tan δ = G″/G′) of control dough sample and dough with OSA starch I (**a**), II (**b**), and III (**c**) with a different substitution level of base starches. OSA I starch—waxy corn starch sodium octenyl succinate; OSA II starch—pregelatinized waxy corn starch sodium octenyl succinate; OSA III starch—hydrolyzed and spray-dried waxy corn starch sodium octenyl succinate.

Changes in the tangent value of the phase shift angle (tan δ = G″/G′) relative to angular frequency for tested gluten-free dough samples with different OSA starch preparations are shown in Figure 2. In the case of OSA I starch (Figure 2a), due to a proportional increase in the value of G′ and G″, the values of the tan δ were not significantly different from each other and remained constant in relation to the angular frequency. The presence of OSA II starch in the dough (Figure 2b) clearly affected the decrease in tan δ value especially below 10 rad/s, which indicates a clear strengthening of the dough structure. On the other hand, the presence of OSA III starch (Figure 2c) resulted in an increase in the tan δ value, which was the greater the larger was the share of the additive, especially in the area of higher angular frequency values, indicating a significant weakening of the structure. These observations are consistent with the tan δ values at 1 Hz frequency (Table 1), which confirm that the doughs tested have characteristics of weak gels. The presence of OSA I or OSA III starch at lower concentrations did not have a significant effect on tan δ values at 1 Hz. On the other hand, the introduction of OSA II starch into the recipe significantly lowered the tan δ values at 1 Hz, indicating strengthening of the dough structure.

**Figure 3:**
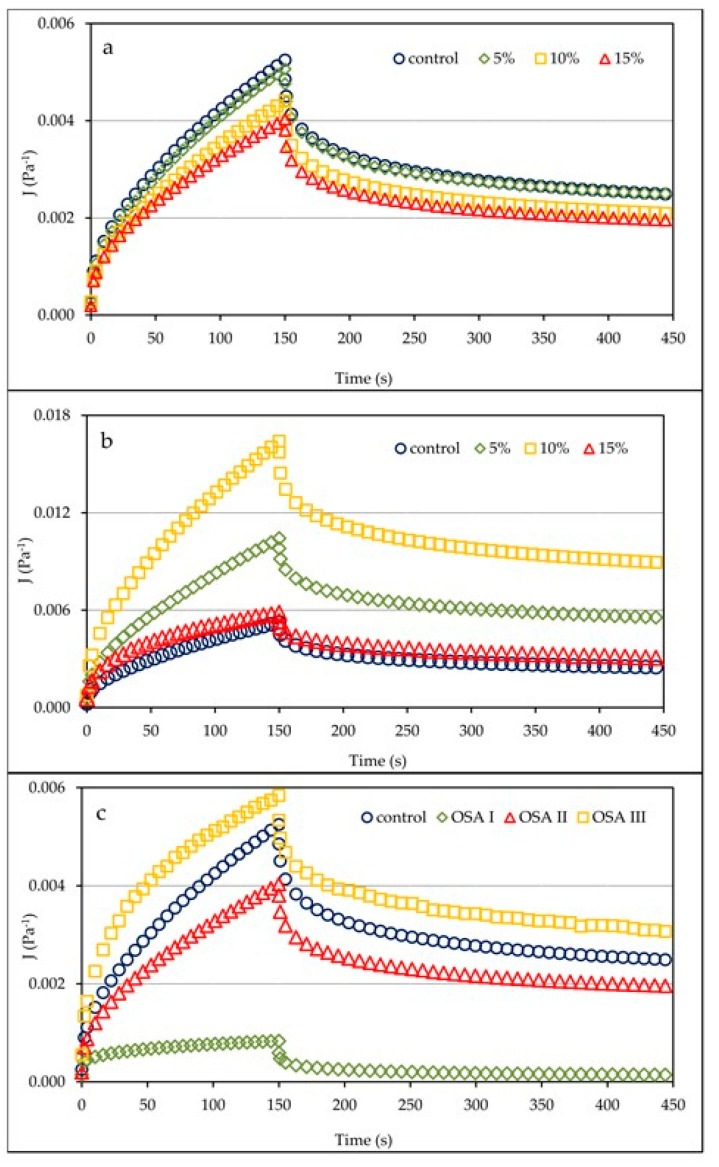
Creep and recovery curves of control dough sample and dough with OSA starch I (**a**) and III (**b**) with different substitution level of base starches and respective plots of all OSA starches with the highest substitution level (15%) of base starches (**c**). OSA I starch—waxy corn starch sodium octenyl succinate; OSA II starch—pregelatinized waxy corn starch sodium octenyl succinate; OSA III starch—hydrolyzed and spray-dried waxy corn starch sodium octenyl succinate.

Examples of creep and recovery curves, as a dependence of compliance on time, for control and OSA starch supplemented dough are shown in Figure 3. As with the sweep frequency test (Figure 1), the presence of individual OSA starch preparations had a varying effect on the compliance values, reflecting the susceptibility of the material to an applied constant stress. In the case of OSA I (Figure 3a), which was only esterified, the introduction of this starch into the dough formulation resulted in a small, but clear, decrease in compliance over time when replacing the base starch in amounts of 10% and 15%. On the other hand, the starch preparation of OSA II caused a very large decrease in the compliance values in time, but the variation in the amount of preparation introduced was small (curves not shown). This has the effect of strengthening the structure of the dough due to the introduction of starch with high swelling capacity. On the other hand, the introduction of the starch preparation OSA III to the recipe caused a clear increase in the dough susceptibility to deformation in time. The greatest influence was exerted by a starch replacing of 10% (Figure 3b). These observations are confirmed by the curves in Figure 3c showing the effect of different OSA starch preparations on the same starch replacing the level of 15% on the dough’s compliance to stress. The presence of OSA starch III increased the susceptibility to stress, confirming a weakening of the dough structure, which is the result of the introduction of starch polymers with a reduced molecular weight and less swelling capacity. On the other hand, the starch preparations OSA I and II influenced the decrease in susceptibility to applied stress, which was particularly evident for the starch preparation OSA II.

These observations are confirmed by the values of the parameters of the Burgers model used to describe the experimental curves (Table 2). The presence of OSA I starch in the formulation influenced the reduction of instantaneous (J_0_) and viscoelastic (J_1_) compliance values, reflecting the elastic and viscoelastic characteristics of the material, although the effect was significant (*p* < 0.05) only for higher concentrations of the starch formulation. A significantly greater effect on the reduction of J_0_ and J_1_ susceptibility values was observed when the OSA II formulation was used, although the statistical variation within the additive level alone was small (Table 2). On the other hand, the introduction of the OSA III starch formulation significantly (*p* < 0.05) increased the susceptibility to applied stress, although without a clear trend, as the highest values of J_0_ and J_1_ compliance were observed in the sample with 10% of the formulation. In the case of retardation time (Table 2) which characterizes the delayed elastic response of a viscoelastic material to an applied stress, the presence of OSA I starch had no significant effect on its values, while the other two formulations at a replacement level of 10% and 15% significantly (*p* < 0.05) affected the decrease for OSA II and the increase for OSA III in the values of this parameter. The values of zero shear viscosity, which is the viscosity extrapolated to zero shear, changed over time in an opposite way to compliance. A small increase in the value of this parameter was recorded for the OSA II starch preparation, which was significant (*p* < 0.05) only for the highest concentration. The introduction of OSA II starch resulted in a significantly higher increase in zero shear viscosity values, whereas for OSA III starch, a significant decrease in this parameter was observed.

Although the methods used to assess the rheological characteristics of dough are based on different foundations, the results obtained using them correlate significantly (*p* < 0.05) with each other. Such a significant negative linear correlation was observed between the values of instantaneous compliance J_0_ and the parameters of equations describing sweep frequency test K′ and K” (r = −0.79 and −0.82, respectively) and viscoelastic compliance J_1_ and constants K′ and K” (r = −0.79 and −0.75, respectively). Furthermore, viscoelastic compliance correlated significantly positively with tan δ values (r = 0.65). In contrast, zero shear viscosity values correlated positively with K′ (r = 0.65) and negatively with tan δ (r = −0.94), respectively.

The viscosity curves of the control dough and samples with OSA starch formulations confirmed that these systems exhibit the characteristics of shear-thinning non-Newtonian liquids (curves not shown), confirming previous observations [5]. The values of the parameters of the power model used to describe the experimental curves are summarized in Table 3. The values of the consistency index indicating the initial viscosity of the dough increased due to the introduction of OSA I starch into the recipe, with a significant (*p* < 0.05) increase observed for the highest concentration of the starch preparation. At the same time, the values of the flow behavior index decreased, indicating a decrease in pseudoplasticity, but the changes were insignificant. On the other hand, the introduction of OSA II starch into the recipe resulted in a significant increase in the consistency coefficient values and a decrease in the flow behavior index values compared to the control sample (Table 3). In the case of OSA III starch, a significant (*p* < 0.05) decrease in the consistency coefficient was observed, with a simultaneous increase in the flow behavior index, indicating a decrease in the viscosity of the dough. 

The results obtained from the viscosity curves are consistent with the results from the small deformation tests. The changes in apparent viscosity during flow, and hence the values of the consistency index, correlate with the changes in the values of the storage and loss moduli, compliance, and zero shear viscosity. This is confirmed by the values of linear correlation coefficients, which indicate a significant (*p* < 0.05) correlation between the consistency coefficient K and the parameters K′ and K” (r = 0.92 and r = 0.83) as well as between the consistency index K and the instantaneous compliance J_0_, viscoelastic compliance J_1_ (r = −0.81 and r = −0.81), and zero shear viscosity η_0_ (r = 0.86).

Since in dough-type systems based on a concentrated starch suspension, the starch is present in granular form, one of the important factors affecting the structure and characteristics of the dough is the swelling of the starch granules. With a constant content of other structure-forming components, in this case guar gum and pectin, and water, the ability of starch to swell seems to be a key factor influencing rheological characteristics of such a dough. Dapčević-Hadnađev et al. [14] found that the effect of introducing OSA starch preparations into a dough recipe depends on their type. Physically unmodified OSA starch causes an increase in water absorption and dough elasticity. On the other hand, pregelatinized OSA starch, despite elevating water absorption, causes an increase in susceptibility to deformation and an increase in elasticity. Hydrolyzed OSA starch, on the other hand, influences a decrease in water absorption, also causing an increase in the susceptibility of the dough to deformation. In addition, Dapčević-Hadnađev et al. [11] observed that the pregelatinized preparation causes an increase in the water absorption of gluten-free dough based on rice starch with added hemp protein. According to Pojić et al. [16], the OSA starch absorbs water to a greater extent below the pasting temperature improving the dough yield, while at the same time making it more resistant to deformation during the first phase of baking, which can be explained by strengthening of walls surrounding gas bubbles in the dough. On the other hand, volume reduction occurs to a lesser extent during the second baking phase due to the higher stiffness of the dough. Akram et al. [17] investigating the effect of different chemically modified starches on dough characteristics found that the addition of OSA starch had the greatest effect on increasing the viscosity of wheat flour suspension during pasting.

### 2.2. Bread Characteristics

The presence of individual OSA starch preparations also influenced the baking properties by changing the rheological characteristics of the dough. The basic parameters characterizing the physical properties of the control sample and bread based on dough in which some starch was replaced by OSA starch preparations have been presented in Table 4. The introduction of the OSA I preparation, i.e., starch subjected only to esterification, to the recipe resulted in a significant (*p* < 0.05) increase in bread volume in comparison with the control sample, to the greatest extent at the highest share of the modified preparation. The share of this starch in the recipe resulted simultaneously in a significant (*p* < 0.05) increase in porosity and in the presence of pores > 5 mm in size. At the same time, the presence of this starch reduced the pore density. The other two OSA starch preparations, physically modified after esterification, affected the characteristics of the bread in a differentiated manner, depending on their amount in the dough (Table 4). The lowest addition of the OSA II starch preparation had no significant effect on the volume of bread, whereas higher concentrations of this starch significantly decreased the value of this parameter. At the same time, the presence of pregelatinized starch caused a significant (*p* < 0.05) increase in porosity and the number of pores > 5 mm in size, with a simultaneous decrease in pore density in comparison with the control sample. In turn, the introduction of the OSA III starch preparation into the recipe resulted in differential effects on bread volume (Table 4). Its lowest proportion decreased the volume, while the highest caused an increase in the value of this parameter. OSA III starch also caused an increase in porosity and the presence of large pores, which was parallel to the proportion of this preparation in the dough. A simultaneous decrease in pore density was also adequate to the share of the spray dried starch in the recipe.

With a constant supply of low molecular weight carbohydrates as substrates for yeast, a key factor affecting the volume of bread is its ability to retain fermentation gases. Many authors point out that the introduction of OSA starch preparations has a beneficial effect on the volume of bread. According to Dapčević-Hadnađev et al. [14], the introduction of OSA starch to bread formulation results in a rise of its volume and average pore size. This trend was confirmed in subsequent studies [15]. In addition, Dapčević-Hadnađev et al. [11] found that the presence of OSA pregelatinized starch increases the volume of gluten-free bread. On the other hand, Pojić et al. [18] found that the addition of OSA starch significantly affects the bread volume, average pore size, and crumb density and hardness, but the direction of these changes also depended on the presence of other tested ingredients i.e., gluten and xylanase. On the other hand, Balic et al. [19] found that replacing fat in wheat bread with OSA starch preparation increased the loaf volume but had no significant effect on pore size and cell wall thickness. Akram et al. [17] investigating the effect of different chemically modified starches on bread properties observed that the addition of OSA starch has the greatest effect on increasing the volume of wheat bread. In addition to the type of OSA starch preparation, the amount of modified starch introduced in the recipe seems to be an important factor. In the case of OSA I starch preparation subjected only to esterification, its introduction into the dough results in a strengthening of its structure and a decrease in its susceptibility to stress (Table 2). This increases the ability of the dough to retain fermentation gases, having a beneficial effect on the increase in bread volume (Table 4). On the other hand, OSA II starch subjected simultaneously to esterification and recleavage, characterized by much higher water absorption capacity, causes a significant decrease in dough susceptibility to deformation (Table 2) and thus its elasticity, limiting the possibility of structure to retain fermentation gases. It results in a decrease of bread volume, especially at higher concentrations of added preparation (Table 4) and indicates that the maximum share of this modifier in the recipe should not exceed 5%. On the other hand, starch preparation OSA III causes an increase of dough susceptibility to deformation (Table 2), which hinders the retention of fermentation gases; therefore, a (Table 4) higher level of substitution of base starches in the recipe, 15%, is necessary to maintain appropriate bread volume.

### 2.3. Crumb Texture

**Figure 4:**
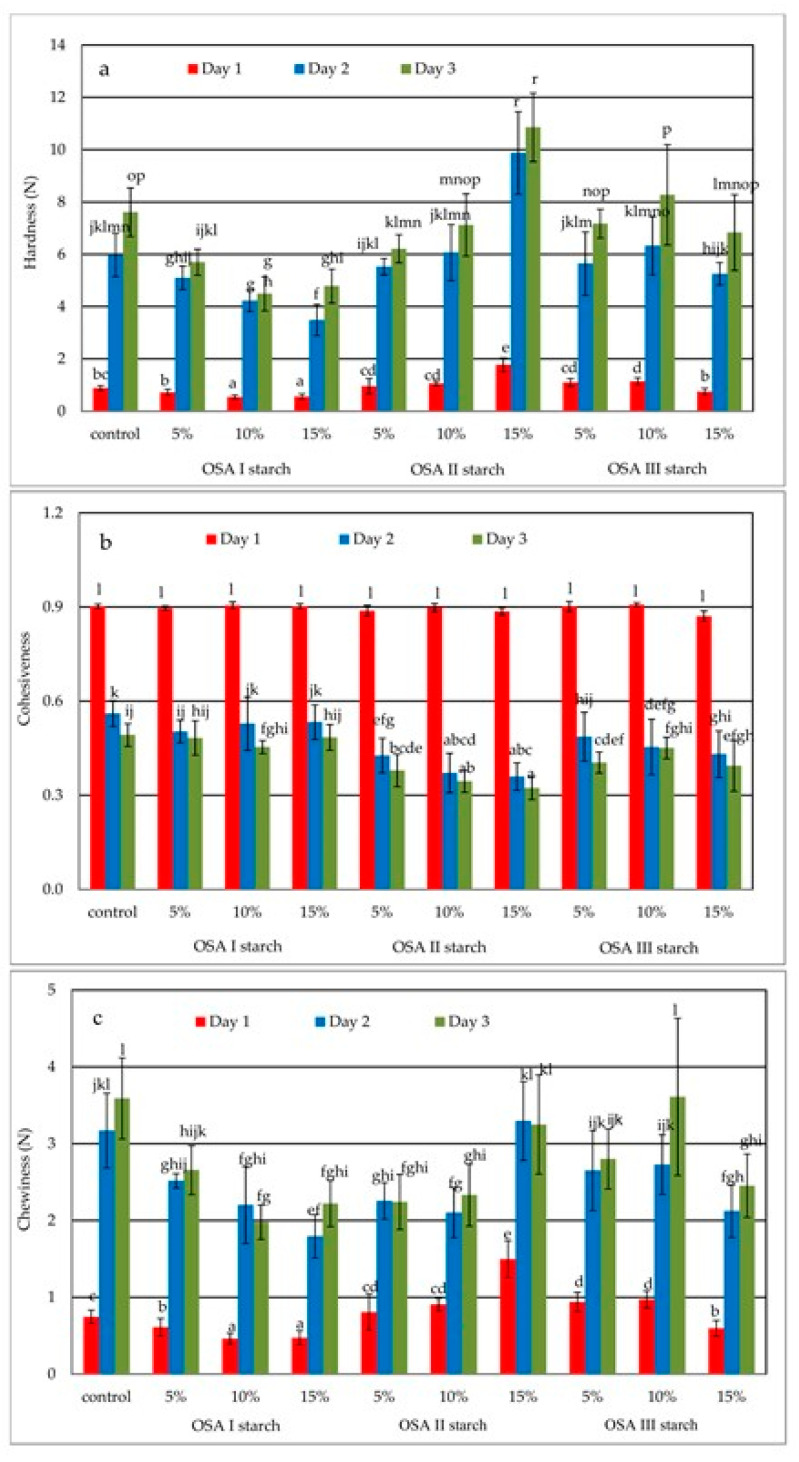
Effect of storage time on the texture parameters: hardness (**a**), cohesiveness (**b**), and (**c**) chewiness. OSA I starch—waxy corn starch sodium octenyl succinate; OSA II starch—pregelatinized waxy corn starch sodium octenyl succinate; OSA III starch—hydrolyzed and spray-dried waxy corn starch sodium octenyl succinate. a–l: Mean values assigned this same letter are non-significantly different at a 0.05 level of confidence.

The textural properties of bread to a large extent determine its acceptance by consumers. During the storage of bread, especially gluten-free starch-based bread, a number of structural changes occur that result in reduced product acceptability. These changes are mainly associated with water migration from crumb to crust and crumb hardening resulting, among others, from starch retrogradation. Various types of additives or ingredients that control water retention and/or reduce starch retrogradation are used to limit these adverse phenomena [1,5,23]. One of the methods to monitor structural changes in the crumb is to measure time-varying parameters characterizing texture. The values of the determined texture parameters i.e., hardness, cohesiveness, and chewiness are shown in Figure 4a–c. The differential effect of the introduction of individual OSA starch preparations into the recipe on crumb hardness is shown in Figure 4a. The presence of the OSA I preparation, i.e., starch subjected only to esterification, at 10% and 15% starch replacement level in the bread recipe had a significant (*p* < 0.05) effect on reducing crumb hardening compared to the control sample on the first and second days of storage. However, on the third day of storage, all levels of OSA I starch addition reduced crumb hardening relative to the control sample. The introduction of OSA II starch preparation in the recipe at 5% and 10% starch replacement level had no significant effect on crumb hardness, but the highest 15% addition caused a significant increase in crumb hardness on each day of analysis. In contrast, OSA III starch preparation had no significant effect on bread crumb hardness compared to the control sample. A three-factor analysis of variance showed that the type of additive and time significantly (*p* < 0.05) affected crumb hardness values, while the level of additive alone had no significant effect (*p* = 0.14). For the combination of factors, only the interaction of time and level had an effect on the values of this parameter. Moreover, a linear negative relationship (r = −0.94) between bread volume and crumb hardness, already known from the literature, was observed. Another parameter determined in the texture evaluation was cohesiveness, which is related to the ability of the particles forming the material to interact with each other. In the case of bread crumb, the decrease of cohesiveness e.g., during storage, causes an increased tendency to crumbling, which negatively affects the consumer acceptance of such a product. The crumb cohesiveness results of the tested samples are shown in Figure 4b. The proportion of OSA starch in the recipe had no significant effect on the cohesiveness values on the day of baking, regardless of the level of addition itself. On the first day of storage, the values of this parameter decreased rapidly due to crumb processes associated with starch retrogradation and bread aging. The effect of the level of OSA starch addition was limited here, but the introduction of OSA II, i.e., additionally pregelatinized starch, to the recipe caused a significantly greater decrease in consistency compared to the control sample. On the third day of storage, only the presence of OSA I starch did not cause an unwanted decrease in consistency values relative to the control sample. For the other two formulations, the decrease was pronounced and greater for the OSA II starch formulation, although the level of additive alone was of limited but significant (*p* = 0.025) importance here. Since chewiness and hardness values are correlated (r = 0.99); the effect of OSA starch preparations on chewiness (Figure 4c) was similar to that on hardness. There was a significant effect of OSA I starch and OSA II starch at 5% and 10% and OSA III starch preparation at the highest proportion on the chewiness values compared to the control sample. A three-factor analysis of variance showed that the additive type and time significantly (*p* < 0.05) affected crumb chewiness values, while additive level alone had no significant effect (*p* = 0.60). For combinations of factors, only the interaction of time and type of starch affected the values of this parameter.

An important factor having a key influence on the correct and acceptable for consumers texture parameters is the ability of dough to retain fermentation gases causing an increase in crumb volume and porosity, which also results in a decrease in its hardness. The addition of OSA starch preparations may have a beneficial effect on decreasing the crumb hardness and increasing the crumb softness. Dapčević-Hadnađev et al. [14] found that the addition of pregelatinized OSA starch has a beneficial effect on texture, creating a softer crumb while increasing bread moisture content and loaf volume. The improvement of textural properties of wheat breads with the addition of OSA starch preparations was later confirmed by Dapčević-Hadnađev et al. [15,24]. In addition, Pojić et al. [16] found a decrease in the crumb hardness of barley bread caused by the addition of OSA starch preparations. On the other hand, Akram et al. [17] while studying the effect of chemically modified starches on bread characteristics found that the crumb of bread containing OSA starch had the lowest hardness both after baking and during storage. The results obtained during the present study (Figure 4) partly confirm the literature data, indicating at the same time that not only the type of OSA starch preparation but also its quantity have an effect on the textural characteristics of the crumb. A too high share of pregelatinized OSA II starch, due to its excessive reinforcement of dough structure and limited ability to retain fermentation gases, apart from decreasing loaf volume, causes an unwanted increase in crumb hardness (Figure 4a).

### 2.4. Thermal Properties

**Figure 5:**
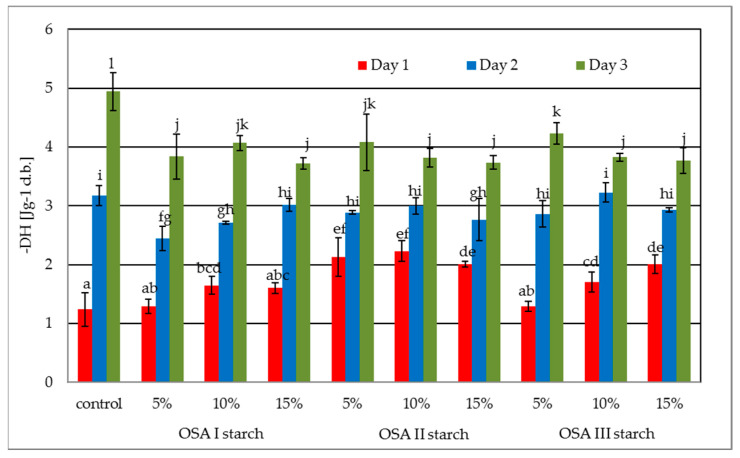
Effect of storage time on the enthalpy of amylopectin melting after the retrogradation of control gluten-free bread and breads with OSA starches. OSA I starch—waxy corn starch sodium octenyl succinate; OSA II starch—pregelatinized waxy corn starch sodium octenyl succinate; OSA III starch—hydrolyzed and spray-dried waxy corn starch sodium octenyl succinate. a–l: Mean values assigned this same letter are non-significantly different at a 0.05 level of confidence.

During the heating of the bread crumb, the decomposition peak of recrystallized amylopectin is present in the thermograms, the area of which corresponds to the enthalpy of this transition. It was found that both the type of preparation and the storage time significantly (*p* < 0.001) influenced the changes in the enthalpy value of the transition related to the decomposition of recrystallized amylopectin. However, the level of the additive alone had no significant effect in this case (*p* = 0.09). In addition, combinations of individual factors had a significant (*p* < 0.05) effect on enthalpy values. In general, enthalpy values increased with increasing storage time regardless of sample type (Figure 5).

Immediately on the day of baking, the presence of the starch preparation OSA II in the recipe increased the enthalpy values of the transition, which may be related to the excessive strengthening of the dough structure and the reduction of the capacity to retain fermentation gases. This also correlates with an increase in crumb hardness caused by the presence of starch preparation OSA II (Figure 4a). In contrast, the introduction of the other two preparations had no significant effect. On the second day of storage, the OSA I starch at a replacement level of 5% and 10% caused a significant decrease in enthalpy values compared to the control sample. On the other hand, on the third day of storage, the presence of all types of OSA starch preparations, irrespective of their concentration, caused a significant decrease in enthalpy values, indicating the possibility to limit the long-term retrogradation of starch, especially amylopectin, which is associated with staling and a decrease in bread quality.

## 3. Materials and Methods

### 3.1. Materials

The research material was corn starch (Bezgluten, Posądza, Poland), potato starch (Pepees S.A., Łomża, Poland), OSA I starch (C*EmTex 06369-waxy corn ester-starch sodium octenyl succinate), OSA II starch (C*EmTex 12688-pregelatinized waxy corn ester-starch sodium octenyl succinate), OSA III starch (C*Emcap 12635-hydrolyzed and spray-dried waxy corn ester-starch sodium octenyl succinate) (Cargill, Poland), guar gum (Lotus Gums & Chemicals, Jodhpur, India), high methylated apple pectin (WEJ-4, esterification degree > 70%, particle size 95% <350 µm (Pektowin, Poland)), freeze-dried yeast (S.I. Lesaffre, France), rapeseed oil Kujawski (ZT Kruszwica, Poland), sugar, salt (purchased from a local supermarket), and water.

### 3.2. Methods

#### 3.2.1. Gluten-Free Dough Formulation

The basic control bread recipe was based on our earlier research [5]. The dough used to bake gluten-free bread was obtained from the following ingredients and additives: corn starch and potato starch (mixed in a ratio of 4:1) 690 g, freeze-dried yeast 34.5 g, vegetable oil 20.7 g, sucrose 13.8 g, salt 11.5 g, pectin 11.5 g, guar gum 11.5 g, and water 656 g. Part (5%, 10% or 15% i.e., 34.5, 69.0, or 103.5 g) of starch mixture, resulting from preliminary research, was replaced by the same amount of OSA starch preparations.

#### 3.2.2. Rheological Properties of the Dough

The rheological properties of the dough were characterized at 25 ± 0.5 °C using the MARS II oscillating rheometer (ThermoFisher Scientific, Waltham, MA, USA) equipped with a parallel plate system (diameter 35 mm, gap size 1 mm). Samples of the dough (without yeast) were placed in the measuring system of the rheometer, protected against evaporation with paraffin oil, and left for 5 min to relax stresses and stabilize the temperature.

The mechanical spectral was determined in the linear viscosity range at a constant deformation amplitude of 0.1% in the angular frequency range of 1–100 rad/s. Experimental data were described by power equations:

(1) $$G^{\prime }\left(\omega \right)=K^{\prime }\cdot \omega^{n^{\prime }}$$

(2) $$G^{\prime \prime }\left(\omega \right)=K^{\prime \prime }\cdot \omega^{n^{\prime \prime }}$$

where *G*′—storage modulus (Pa), *G″*—loss modulus (Pa), *ω*—angular frequency (rad/s), *K*′, *K*”, *n*′, and *n″*—constants.

Creep and recovery tests were performed at constant strain in creep phase τ_0_ = 2 Pa during 150 s. The recovery phase continued for 300 s. Experimental data were described using the Burgers model:

(3) $$J(t)=J_{0}+\frac{t}{\eta_{0}}+J_{1}\cdot (1-\exp^{-t/\lambda_{ret}})$$

(4) $$J(t)=\frac{t_{1}}{\eta_{0}}-J_{1}\cdot (1-\exp^{t_{1}/\lambda_{ret}})\cdot \exp^{-t/\lambda_{ret}}$$

where *J*—compliance (Pa^−1^), *J*_0_—instantaneous compliance (Pa^−1^), *J*_1_—viscoelastic compliance (Pa^−1^), *η*_0_—zero shear viscosity (Pa∙s), *λ_ret_*—retardation time (s), and *t*_1_—the time after which the stress was removed (s).

The viscosity curves were determined at the controlled shear rate in the range 1–10 s^−1^. Experimental data were described by the power equation:

(5) $$\eta_{ap}=K\cdot {\dot{\gamma }}^{n}$$

where *η_ap_*—apparent viscosity (Pa·s), *K*—consistency coefficient (Pa·s^n^), $\dot{\gamma }$—shear rate (s^−1^), and *n*—flow behavior index.

#### 3.2.3. Dough Preparation and Bread Baking

After weighing, all dough ingredients were mixed for 8 min (Laboratory Spiral Mixer SP 12, Diosna, Germany). The resulting dough was fermented for 15 min (35 °C, 80% relative humidity) and stirred again for one minute. Then, 250 g of dough was weighed into greased metal forms. The final fermentation was carried out within 20 min under conditions as stated earlier. The bread was baked within 30 min at 230 °C (MIWE Condo electric oven type CO 2 0608, MIWE GmbH, Arnstein, Germany). Once removed from the oven, the bread was cooled for two hours at ambient temperature (around 21 °C) and used for further analysis. Each dough was made in duplicate based on individual recipes; five loaves were baked in each repetition. In order to assess changes in texture parameters during storage, some samples were packed in polyethylene bags and stored at 22 ± 2 °C.

#### 3.2.4. Bread Characteristics

The volume of bread was measured using the Volscan 600 (Stable Micro Systems, Godalming, UK). Image analysis was performed for 1 cm thick samples taken from the inner parts of each loaf that were scanned with the Plustek S-12 flatbed scanner. Recorded images were analyzed using ImageJ v. 1.44p. Porosity, pore density, and average pore size were assessed.

Texture profile analysis (TPA) of bread crumb was performed using the TA-XT2plus texture analyzer (Stable Micro Systems, Godalming, UK) at a compression rate of 5 mm/s. A cylindrical sample of bread crumb with a diameter 1 cm and height 2 cm was compressed to achieve 50% deformation with the P/35 aluminum cylindrical probe in two cycles. The resulting hardness, cohesiveness, and chewiness values were used as indicators for the changes in crumb texture during storage. Calculations were performed using Texture Exponent (Stable Micro Systems, Godalming, UK). Analyses were carried out 2, 24, and 48 h after baking.

The thermal characteristics of the bread crumb were obtained using the DSC 204F1 Phoenix differential scanning calorimeter (Netzsch GmbH, Selb, Germany). On each day of analysis, after 2, 24, and 48 h after baking, crumb samples weighing 15 mg of were taken from the center of the loaf, hermetically sealed in aluminum pans, and heated in a calorimeter from 20 to 100 °C at a rate of 10 °C/min. The empty aluminum pan was used as a standard. Thermal transition temperature and enthalpy were calculated using the Proteus Analysis software (Netzsch GmbH, Selb, Germany). Enthalpy values were expressed in J/g of d.b.

#### 3.2.5. Statistical Analysis

In order to assess the significance of the differences between the averages, the data were subjected to a single- and two-factor analysis of variance, and the significance of the differences between the averages was determined by the Duncan’s test at a significance level of 0.05. A three-factor ANOVA was used to determine the effect of the type of OSA starch, the level of its addition, and the storage time on texture parameters and enthalpy of the recrystallized amylopectin decomposition. The relationship between the resulting parameters was assessed using Pearson’s correlation coefficient values, the significance of which was checked using the Student’s *t*-test. Calculations were performed using Statistica 12.0 (StatSoft Inc., Tulsa, OK, USA).

## 4. Conclusions

On the basis of the results, it was found that the analyzed dough samples behaved as weak gels and the values of moduli G′ and G″ significantly depended on the angular frequency. The introduction of OSA starch preparations modified the dough structure in a different way, influencing changes in its rheological characteristics and susceptibility to stress. The key factor here seems to be the ability of these preparations to absorb water. The OSA preparation subjected only to esterification strengthened the structure of the dough, influencing the decrease of its compliance to stress. This increases the possibility of the dough to retain fermentation gases, having a positive effect on the increase in bread volume. The preparation of pregelatinized OSA starch, on account of its high water absorption capacity, caused significant strengthening of the structure and a rapid decrease in susceptibility to stress. However, a too high share of pregelatinized OSA starch, through too high strengthening of the dough structure and limited ability to retain fermentation gases, caused a decrease in the volume of loaves and an increase in crumb hardness. On the other hand, hydrolyzed and spray-dried OSA starch significantly increased the dough susceptibility to stress, weakening its structure. This resulted in a decrease of bread volume at a lower proportion of such preparation. On the last day of the study, a significant decrease in enthalpy of retrograded amylopectin decomposition values was observed in all breads containing the tested OSA starch preparations, regardless of their amount. This indicates the possibility of reducing the retrogradation of amylopectin responsible for crumb hardening during bread storage. In terms of the quality of the bread related to the increase in the volume of the loaves and the delay in hardening of the crumb during storage compared to the control sample, the preparation of waxy corn starch sodium octenyl succinate showed the best effect.

## Figures and Tables

**Table 1:** Parameters of the power-law functions describing the dependence of storage and loss moduli on the angular frequency and tan δ at frequency of 1 Hz of control gluten-free dough and dough with OSA starch addition.

Sample		K’ (Pa·s^n^′^^)	n’ (-)	R^2^>	K” (Pa·s^n^″^^)	n″ (-)	R^2^>	tan *δ* at 1 Hz
**Control GFB**		1353.6 ± 33.8 ^d^	0.204 ± 0.001 ^c^	0.999	509.4 ± 13.9 ^d^	0.203 ± 0.002 ^b^	9.956	0.426 ± 0.003 ^c^
OSA I	5%	1356.0 ± 145.0 ^d^	0.206 ± 0.004 ^c^	0.999	521.0 ± 47.5 ^d^	0.197 ± 0.005 ^ab^	0.959	0.436 ± 0.005 ^c^
	10%	1634.8 ± 167.2 ^e^	0.206 ± 0.006 ^c^	0.996	621.8 ± 67.5 ^e^	0.196 ± 0.006 ^ab^	0.952	0.432 ± 0.004 ^c^
	15%	1826.2 ± 213.5 ^e^	0.203 ± 0.004 ^c^	0.999	699.0 ± 71.1 ^f^	0.188 ± 0.006 ^a^	0.950	0.432 ± 0.009 ^c^
OSA II	5%	3899.1 ± 226.9 ^g^	0.149 ± 0.003 ^a^	0.987	964.6 ± 58.5 ^g^	0.198 ± 0.006 ^ab^	0.933	0.287 ± 0.004 ^b^
	10%	3649.3 ± 317.9 ^g^	0.147 ± 0.004 ^a^	0.997	757.2 ± 61.9 ^f^	0.253 ± 0.008 ^d^	0.970	0.235 ± 0.005 ^a^
	15%	2540.8 ± 296.7 ^f^	0.169 ± 0.009 ^b^	0.992	545.2 ± 45.9 ^d^	0.309 ± 0.006 ^f^	0.989	0.232 ± 0.008 ^a^
OSA III	5%	784.9 ± 46.7 ^c^	0.209 ± 0.002 ^c^	0.995	294.6 ± 14.8 ^c^	0.226 ± 0.008 ^c^	0.947	0.440 ± 0.012 ^c^
	10%	436.9 ± 30.9 ^b^	0.213 ± 0.009 ^c^	0.998	155.7 ± 13.2 ^b^	0.285 ± 0.021 ^e^	0.962	0.444 ± 0.018 ^c^
	15%	214.5 ± 9.2 ^a^	0.244 ± 0.024 ^d^	0.991	84.6 ± 8.6 ^a^	0.384 ± 0.011 ^g^	0.988	0.534 ± 0.032 ^d^
One-way ANOVA-p		<0.001	<0.001		<0.001	<0.001		<0.001
Two-way ANOVA-p								
Factor A (Type)		<0.001	<0.001		<0.001	<0.001		<0.001
Factor B (Level)		<0.001	<0.001		<0.001	<0.001		<0.001
Factor A × Factor B		<0.001	<0.001		<0.001	<0.001		<0.001

^a^–^g^: Mean value of three replication ± standard deviation. Mean values assigned the same letter in particular columns are non-significantly different at a 0.05 level of confidence.

**Table 2:** Parameters of Burgers model of control gluten-free dough and dough with OSA starch addition.

Sample		J_0_ × 10^3^ (Pa^−1^)	J_1_ × 10^3^ (Pa^−1^)	λ_ret_ (s)	η_0_ × 10^−3^ (Pa·s)	R^2^>
**Control GFB**		1.06 ± 0.03 ^d^	1.84 ± 0.16 ^d^	62.6 ± 5.0 ^bc^	58.6 ± 2.55 ^cd^	0.986
OSA I	5%	0.96 ± 0.10 ^cd^	1.73 ± 0.17 ^d^	59.4 ± 2.1 ^b^	58.53 ± 9.21 ^cd^	0.988
	10%	0.84 ± 0.05 ^c^	1.56 ± 0.12 ^cd^	62.4 ± 5.8 ^bc^	69.88 ± 4.50 ^de^	0.984
	15%	0.83 ± 0.11 ^c^	1.36 ± 0.10 ^c^	61.2 ± 1.4 ^bc^	74.97 ± 8.22 ^e^	0.983
OSA II	5%	0.32 ± 0.03 ^a^	0.36 ± 0.02 ^b^	58.9 ± 2.1 ^b^	757.71 ± 33.45 ^f^	0.989
	10%	0.29 ± 0.03 ^a^	0.25 ± 0.02 ^a^	46.9 ± 3.3 ^a^	1273.26 ± 217.10 ^h^	0.992
	15%	0.41 ± 0.05 ^b^	0.28 ± 0.04 ^a^	41.6 ± 5.9 ^a^	968.49 ± 174.69 ^g^	0.991
OSA III	5%	1.91 ± 0.16 ^e^	3.27 ± 0.37 ^e^	65.0 ± 6.9 ^bc^	26.67 ± 3.15 ^b^	0.986
	10%	3.23 ± 0.31 ^f^	5.08 ± 0.49 ^f^	68.9 ± 5.2 ^bc^	16.51 ± 1.36 ^a^	0.981
	15%	1.77 ± 0.13 ^e^	1.71 ± 0.16 ^d^	71.9 ± 12.0 ^c^	47.93 ± 7.31 ^c^	0.961
One-way ANOVA-p		<0.001	<0.001	<0.001	<0.001	
Two-way ANOVA-p						
Factor A (Type of addition)		<0.001	<0.001	<0.001	<0.001	
Factor B (Level of addition)		0.047	<0.001	0.628	<0.001	
Factor A × Factor B		<0.001	<0.001	0.027	<0.001	

^a^–^g^: Mean value of three replication ± standard deviation. Mean values assigned this same letter in particular columns are non-significantly different at a 0.05 level of confidence.

**Table 3:** Parameters of the power-law functions describing flow properties of gluten-free dough.

Sample		K	n	R^2^>
		(Pa·s^n^)	(-)	
**Control GFB**		529.2 ± 25.2 ^d^	0.216 ± 0.005 ^c^	0.992
OSA I	5%	558.8 ± 26.1 ^d^	0.188 ± 0.009 ^c^	0.990
	10%	572.4 ± 31.9 ^de^	0.192 ± 0.017 ^c^	0.990
	15%	674.7 ± 43.3 ^e^	0.193 ± 0.023 ^c^	0.993
OSA II	5%	909.2 ± 33.4 ^f^	0.132 ± 0.032 ^b^	0.998
	10%	1172.6 ± 74.1 ^g^	0.066 ± 0.006 ^a^	0.998
	15%	1107.3 ± 134.5 ^g^	0.185 ± 0.026 ^c^	0.996
OSA III	5%	289.7 ± 16.6 ^c^	0.294 ± 0.023 ^d^	0.994
	10%	179.9 ± 28.1 ^b^	0.399 ± 0.030 ^e^	0.993
	15%	41.4 ± 4.4 ^a^	0.721 ± 0.042 ^f^	0.998
One-way ANOVA-p		<0.001	<0.001	
Two-way ANOVA-p				
Factor A (type of addition)		<0.001	<0.001	
Factor B (level of addition)		<0.001	<0.001	
Factor A × Factor B		<0.001	<0.001	

^a^–^g^: Mean value of three replication ± standard deviation. Mean values assigned this same letter in particular columns are non-significantly different at a 0.05 level of confidence.

**Table 4:** Bread volume and digital image analysis parameters of control gluten-free bread crumb and bread crumb with OSA starch addition.

Sample		Volume (cm^3^)	Porosity (-)	Cell Density (cm^−2^)	Percentage of Pores > 5 mm (-)
**Control GFB**		600.0 ± 15.12 ^de^	0.395 ± 0.005 ^a^	3.612 ± 0.315 ^f^	0.103 ± 0.005 ^a^
OSA I	5%	630.0 ± 17.73 ^fg^	0.469 ± 0.011 ^def^	2.968 ± 0.267 ^e^	0.138 ± 0.014 ^b^
	10%	640.0 ± 26.73 ^fg^	0.480 ± 0.015 ^ef^	2.628 ± 0.244 ^d^	0.161 ± 0.012 ^cd^
	15%	655.0 ± 17.73 ^g^	0.482 ± 0.012 ^f^	2.516 ± 0.218 ^d^	0.157 ± 0.011 ^bc^
OSA II	5%	613.8 ± 20.66 ^ef^	0.476 ± 0.012 ^ef^	2.224 ± 0.219 ^c^	0.180 ± 0.011 ^d^
	10%	551.3 ± 33.57 ^c^	0.465 ± 0.009 ^de^	1.778 ± 0.108 ^b^	0.212 ± 0.003 ^e^
	15%	465.0 ± 38.91 ^a^	0.461 ± 0.011 ^d^	2.241 ± 0.102 ^b^	0.190 ± 0.033 ^e^
OSA III	5%	523.8 ± 16.06 ^b^	0.415 ± 0.004 ^b^	2.676 ± 0.082 ^d^	0.178 ± 0.004 ^cd^
	10%	581.3 ± 28.00 ^d^	0.439 ± 0.009 ^c^	2.014 ± 0.202 ^bc^	0.219 ± 0.008 ^e^
	15%	637.5 ± 19.09 ^fg^	0.483 ± 0.005 ^f^	1.072 ± 0.066 ^a^	0.259 ± 0.017 ^f^
One-way ANOVA-p		<0.001	<0.001	<0.001	<0.001
Two-way ANOVA-p					
Factor A (Type of Additive)		<0.001	<0.001	<0.001	<0.001
Factor B (Level of Additive)		0.773	<0.001	<0.001	<0.001
Factor A × Factor B		<0.001	<0.001	<0.001	0.003

^a^–^g^: Mean value of six replication ± standard deviation. Mean values assigned this same letter in particular columns are non-significantly different at a 0.05 level of confidence.

## Data Availability

All data is included in the article.
